# Uganda’s experience in Ebola virus disease outbreak preparedness, 2018–2019

**DOI:** 10.1186/s12992-020-00548-5

**Published:** 2020-03-19

**Authors:** Jane Ruth Aceng, Alex R. Ario, Allan N. Muruta, Issa Makumbi, Miriam Nanyunja, Innocent Komakech, Andrew N. Bakainaga, Ambrose O. Talisuna, Collins Mwesigye, Allan M. Mpairwe, Jayne B. Tusiime, William Z. Lali, Edson Katushabe, Felix Ocom, Mugagga Kaggwa, Bodo Bongomin, Hafisa Kasule, Joseph N. Mwoga, Benjamin Sensasi, Edmund Mwebembezi, Charles Katureebe, Olive Sentumbwe, Rita Nalwadda, Paul Mbaka, Bayo S. Fatunmbi, Lydia Nakiire, Mohammed Lamorde, Richard Walwema, Andrew Kambugu, Judith Nanyondo, Solome Okware, Peter B. Ahabwe, Immaculate Nabukenya, Joshua Kayiwa, Milton M. Wetaka, Simon Kyazze, Benon Kwesiga, Daniel Kadobera, Lilian Bulage, Carol Nanziri, Fred Monje, Dativa M. Aliddeki, Vivian Ntono, Doreen Gonahasa, Sandra Nabatanzi, Godfrey Nsereko, Anne Nakinsige, Eldard Mabumba, Bernard Lubwama, Musa Sekamatte, Michael Kibuule, David Muwanguzi, Jackson Amone, George D. Upenytho, Alfred Driwale, Morries Seru, Fred Sebisubi, Harriet Akello, Richard Kabanda, David K. Mutengeki, Tabley Bakyaita, Vivian N. Serwanjja, Richard Okwi, Jude Okiria, Emmanuel Ainebyoona, Bernard T. Opar, Derrick Mimbe, Denis Kyabaggu, Chrisostom Ayebazibwe, Juliet Sentumbwe, Moses Mwanja, Deo B. Ndumu, Josephine Bwogi, Stephen Balinandi, Luke Nyakarahuka, Alex Tumusiime, Jackson Kyondo, Sophia Mulei, Julius Lutwama, Pontiano Kaleebu, Atek Kagirita, Susan Nabadda, Peter Oumo, Robinah Lukwago, Julius Kasozi, Oleh Masylukov, Henry Bosa Kyobe, Viorica Berdaga, Miriam Lwanga, Joe C. Opio, David Matseketse, James Eyul, Martin O. Oteba, Hasifa Bukirwa, Nulu Bulya, Ben Masiira, Christine Kihembo, Chima Ohuabunwo, Simon N. Antara, Wilberforce Owembabazi, Paul B. Okot, Josephine Okwera, Isabelle Amoros, Victoria Kajja, Basnet S. Mukunda, Isabel Sorela, Gregory Adams, Trevor Shoemaker, John D. Klena, Celine H. Taboy, Sarah E. Ward, Rebecca D. Merrill, Rosalind J. Carter, Julie R. Harris, Flora Banage, Thomas Nsibambi, Joseph Ojwang, Juliet N. Kasule, Dan F. Stowell, Vance R. Brown, Bao-Ping Zhu, Jaco Homsy, Lisa J. Nelson, Patrick K. Tusiime, Charles Olaro, Henry G. Mwebesa, Yonas Tegegn Woldemariam

**Affiliations:** 1 Ministry of Health, Kampala, Uganda; 2 Uganda Public Health Fellowship Program, Ministry of Health, Kampala, Uganda; 3 Public Health Emergency Operations Centre, Ministry of Health, Kampala, Uganda; 4 World Health Organisation, Country Office, Kampala, Uganda; 5 World Health Organization – AFRO, Brazzaville, Congo; 6 World Health Organisation, Geneva, Switzerland; 7 Infectious Disease Institute, Kampala, Uganda; 8 African Field Epidemiology Network, Kampala, Uganda; 9 Management Sciences for Health, Kampala, Uganda; 10 Makerere University Walter Reed Project, Kampala, Uganda; 11 East African Public Health Laboratory Network, Kampala, Uganda; 12 Ministry of Agriculture, Animal Industry and Fisheries, Entebbe, Uganda; 13 Uganda Virus Research Institute, Entebbe, Uganda; 14 Uganda National Health Laboratory Services, Ministry of Health, Kampala, Uganda; 15 Ministry of Internal Affairs, Uganda Police Force, Kampala, Uganda; 16 Department for International Development, UKAID, Kampala, Uganda; 17 United Nations High Commissioner for Refugees, Kampala, Uganda; 18 World Food Program, Kampala, Uganda; 19 African Risk Capacity, Kampala, Uganda; 20 United Nations Children’s Fund, Kampala, Uganda; 21 Civil Aviation Authority, Entebbe, Uganda; 22 United States Agency for International Development, Kampala, Uganda; 23 Uganda Red Cross Society, Kampala, Uganda; 24 Medicines San Frontiers, Kampala, Uganda; 25 Intenational Organisation for Migration, Kampala, Uganda; 26 National Center for Emerging and Zoonotic Infectious Diseases, US Centers for Disease Control and Prevention, Atlanta, GA USA; 27 Division of Global Migration and Quarantine, Global Border Health, US Centers for Disease Control and Prevention, Atlanta, GA USA; 28 Global Immunization Division, US Centers for Disease Control and Prevention, Atlanta, GA USA; 29 US Centers for Disease Control and Prevention, Kampala, Uganda

**Keywords:** Ebola, Viral Haemorrhagic fever, Epidemic preparedness, Disease outbreaks, Global Health security, Uganda

## Abstract

**Background:**

Since the declaration of the 10th Ebola Virus Disease (EVD) outbreak in DRC on 1st Aug 2018, several neighboring countries have been developing and implementing preparedness efforts to prevent EVD cross-border transmission to enable timely detection, investigation, and response in the event of a confirmed EVD outbreak in the country. We describe Uganda’s experience in EVD preparedness.

**Results:**

On 4 August 2018, the Uganda Ministry of Health (MoH) activated the Public Health Emergency Operations Centre (PHEOC) and the National Task Force (NTF) for public health emergencies to plan, guide, and coordinate EVD preparedness in the country. The NTF selected an Incident Management Team (IMT), constituting a National Rapid Response Team (NRRT) that supported activation of the District Task Forces (DTFs) and District Rapid Response Teams (DRRTs) that jointly assessed levels of preparedness in 30 designated high-risk districts representing category 1 (20 districts) and category 2 (10 districts). The MoH, with technical guidance from the World Health Organisation (WHO), led EVD preparedness activities and worked together with other ministries and partner organisations to enhance community-based surveillance systems, develop and disseminate risk communication messages, engage communities, reinforce EVD screening and infection prevention measures at Points of Entry (PoEs) and in high-risk health facilities, construct and equip EVD isolation and treatment units, and establish coordination and procurement mechanisms.

**Conclusion:**

As of 31 May 2019, there was no confirmed case of EVD as Uganda has continued to make significant and verifiable progress in EVD preparedness. There is a need to sustain these efforts, not only in EVD preparedness but also across the entire spectrum of a multi-hazard framework. These efforts strengthen country capacity and compel the country to avail resources for preparedness and management of incidents at the source while effectively cutting costs of using a “fire-fighting” approach during public health emergencies.

## Background

A week after declaring the end of the 9th Ebola Virus Disease (EVD) outbreak in Equateur province of the Democratic Republic of Congo (DRC) on July 24th, 2018, the Ministry of Health of the DRC confirmed a new EVD outbreak in the Eastern DRC, North Kivu province on August 1, 2018 [1]. Six health zones, including Beni, Butembo, Oicha, Mabalako, and Musienene in North-Kivu province, and Mandima in Ituri province have since reported confirmed and probable EVD cases. Within a month, 122 EVD cases (91 confirmed, 31 probable), including 82 deaths (67% case-fatality rate) had been reported with an additional nine suspected cases pending laboratory testing for EVD [2]. North Kivu province shares borders with Uganda in the east, and there is frequent cross-border movement due to trade, farming, healthcare, and social activities. In addition, North Kivu and Ituri provinces have been the centre of interethnic clashes for some time now, and harbor over a million displaced persons, with active rebel activities causing prolonged insecurity. Since December 2018, there has been a continuous influx of refugees into Uganda, resulting in a high risk of EVD introduction into the country.

EVD is a severe, often fatal illness in humans. Initial human infection with Ebola virus (EBOV) occurs through contact with an infected animal, such as a fruit bat or non-human primate [3]. The virus spreads from person to person via direct contact with the blood or body fluids of an EBOV-infected symptomatic person or dead body. Entry of the body fluids (urine, saliva, sweat, faeces, vomit, breast milk, vaginal fluid, and semen) through broken skin or mucous membranes in the eyes, nose, or mouth can lead to infection [4].

The spread of Ebola Zaire strain can be effectively prevented through vaccination, as demonstrated by a ring vaccination trial held in Guinea during the 2014 West African EVD outbreak [5]. Guinea was the epicenter and the initial site of the largest-ever EVD outbreak, leading to intense transmission in Guinea as well as the neighbouring countries of Liberia and Sierra Leone. Transmission to neighbouring countries was associated with ill-prepared health systems and poor inter-governmental coordination, factors that led to poor disease surveillance, insufficient infection prevention and control and clinical care. Liberia, Guinea and Sierra Leone had a long history of socio-economic underdevelopment due to civil conflict leading to very weak and severely under-resourced health systems. The outbreak was further exacerbated by infected persons crossing the highly porous borders of the countries involved [5, 6]. As a result, this outbreak caused significant mortality, with reported case-fatality rates of up to 70%.

EVD outbreaks have also occurred in the central African nations of Gabon (1995–96, 2001), the Democratic Republic of Congo (DRC) (1976, 1977, 1995, 2007, 2008, 2012, 2014, 2017, 2018), Sudan(2004), and Uganda (2000, 2007, 2011, 2012) [7, 8]. Uganda’s largest documented EVD outbreak occurred in 2000–2001 in Gulu District, registering 425 cases and 224 deaths [9]. In this paper, we describe Uganda’s experience in preparedness to prevent EVD introduction into the country and limit its spread in case of an outbreak. The approach adopted by the country focused on comprehensively addressing objectives of epidemic preparedness and reponse, including anticipation/prediction so that epidemics do not occur, early detection, and rapid and effective response.

## Methods

The epidemic preparedness process constituted all the activities that were undertaken by MoH and its partners from national to health facility levels to enable readiness to effective response to EVD outbreak in Uganda from August 2018 when EVD outbreak was declared in the DRC to the time of writing this paper. The reports of the National Task Force (NTF) and its subcommittees were reviewed to inform the content of the paper.

## Results

The preparedness efforts included activation of coordination mechanisms, functionalising subcommittees of the NTF, classifying districts by risk level and conducting risk assessment and mapping exercise which informed development of the national EVD response plan. To assess the country readiness, a major simulation exercise was conducted.

### Activation of the PHEOC, NTF, and DTFs

**Fig. 1:**
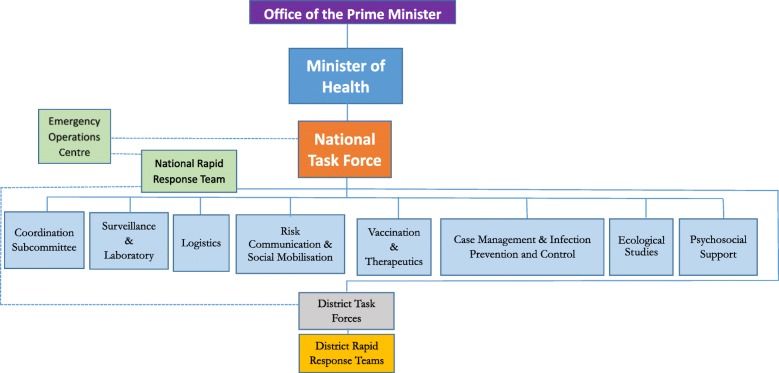
Showing the National Task Force and relationship to its subcommittees, District Task Forces and Rapid Response Teams

The MoH activated the Public Health Emergency Operations Centre (PHEOC), NTF and District Task Forces (DTFs) for coordination of the EVD preparedness. The NTF is multi-sectoral and multi-disciplinary in nature and comprises key ministries, agencies, and departments as well as partners and relevant stakeholders and works through its subcommittees (Fig. 1).

The NTF which is chaired by the Director General of Health Services and co-chaired by WHO, held an urgent meeting on the 2nd of August 2018 to discuss EVD preparedness and prevention of spillover from DRC into Uganda. The NTF developed a multi-agency Incident Management System (IMS) to coordinate preparedness efforts and guide central and field EVD activities. The major IMS components are command and management of preparedness activities, resource management, communication, and information management. The NTF assigned an Incident Management Commander (IMC) and team, made of 6 technical subcommittee team leads encompassing the eleven key WHO EVD preparedness components, including: 1) epidemiological surveillance (contact tracing, capacities at PoEs, and laboratory incorporated); 2) case management and Infection Prevention and Control (IPC) including psycho-social support, waste management and safe and dignified burials; 3) risk communication and community engagement; 4) vaccination, therapeutics, and research; 5) emergency coordination including budgeting and resource mobilisation; and 6) logistics [10]. The IMT was responsible for planning and managing preparedness resources and activities, guiding activity implementation through the DTFs, and reporting back progress from each district to the NTF. After the initial risk assessment, 20 districts bordering North Kivu and Ituri provinces were categorized as high-risk for EVD importation, 10 as moderate-risk, and the remaining districts as low-risk. Through the IMS, all 20 high-risk and 10 moderate-risk districts were supported to formulate and/or reactivate their DTFs to constitute sub-committees for the 6 core preparedness components and coordinate interventions at district level. The DTFs comprised the district political, civic, security, and health leadership as well as technical advisors from different partners working in the districts.

**Fig. 2:**
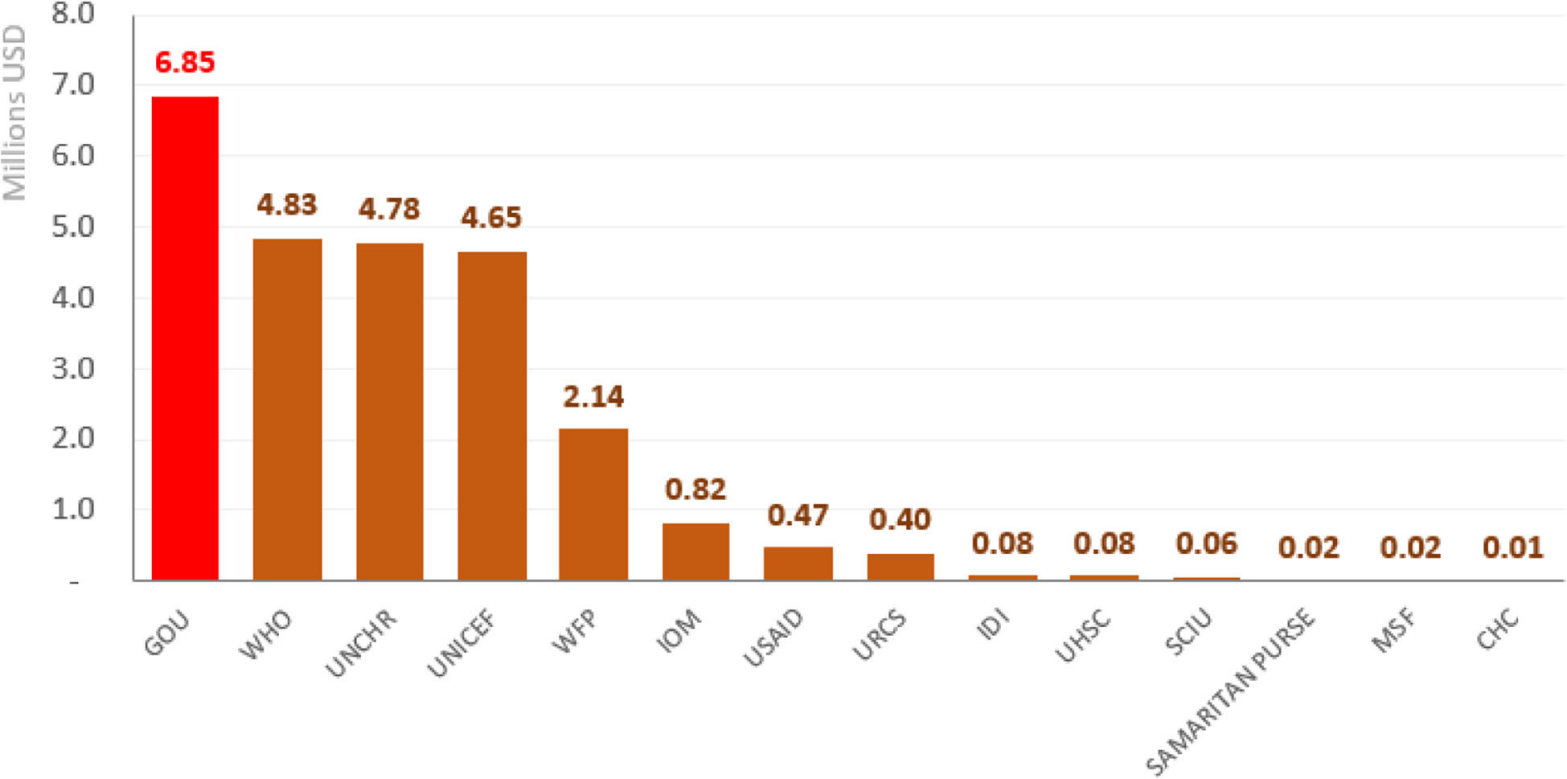
National Budget and Financial Contribution to EVD preparedness by Implementing Partner

**Fig. 3:**
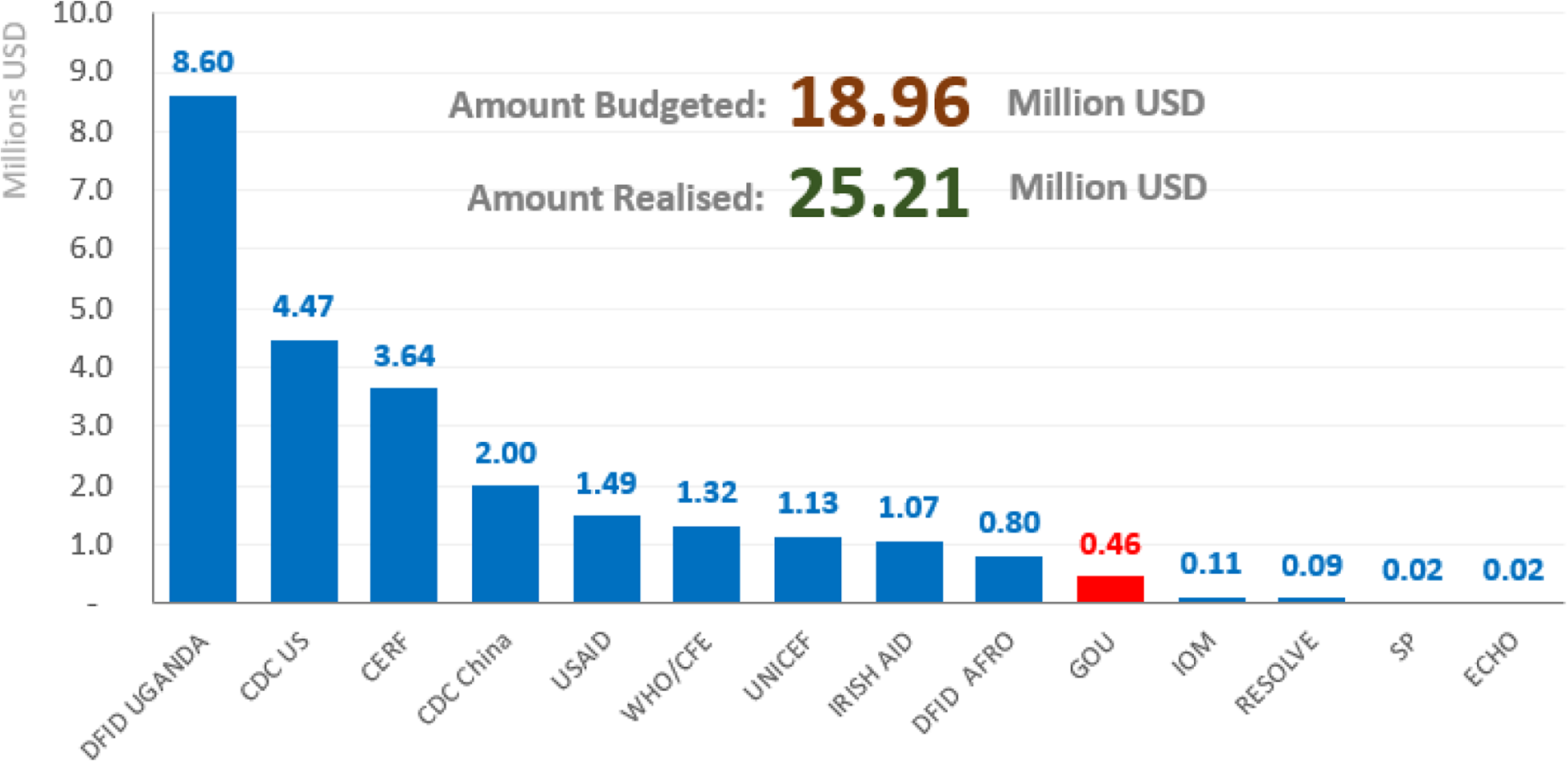
Financial Contribution to EVD preparedness by Donor

As a result of the complexity of the EVD situation in DRC, civil unrest and refugee influx, several national development partners became involved in EVD preparedness. The NTF, with support from WHO, developed a partners’ coordination matrix to avoid duplication of efforts, facilitate identification of preparedness gaps, support monitoring of implementation of the preparedness plan and realise the importance of impact on competing health interests. The matrix specified who should be doing what, where, and when (also known as the 4Ws), as well as the EVD preparedness goal, objectives, strategies, budget, funding sources, funding gaps, donors, and implementing agencies in the respective high-risk districts. Guided by this matrix, the MoH and partner institutions provided district-level financial and technical support for preparedness activities (Figs. 2 and 3). Figure 2 shows total amount of money spent by each implementing partner while Fig. 3 shows the contribution by each donor to the preparedness efforts.

The matrix was updated regularly based on feedback from NTF members. A dashboard was developed and updated promptly to display the financial and technical progress as well as the 4Ws.

### Risk classification of districts

**Fig. 4:**
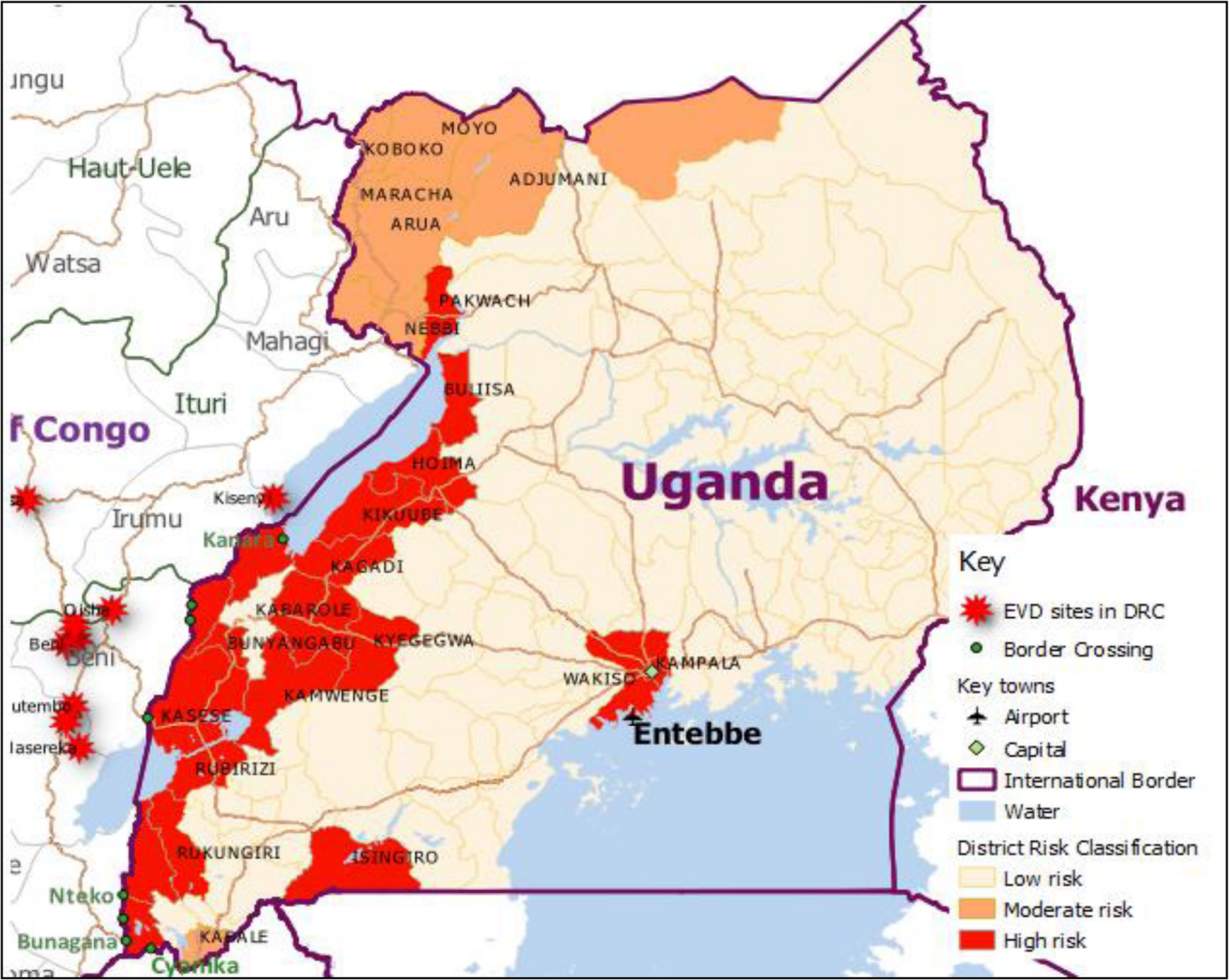
Geographical distribution of the 30 EVD high-risk districts of Uganda, 2018

The NTF classified district EVD transmission risk into 3 categories. Category 1 included 20 high-risk districts that have physical borders, direct routes, and refugee influx from the affected provinces in DRC as well as the central business disticts of Kampala Capital City and Wakiso. Category 2 included 10 moderate-risk districts that have physical borders with DRC but no direct route to EVD-affected regions, and category 3 included low-risk districts in the rest of the country (Fig. 4).

### District rapid response and risk assessments

The NTF deployed the NRRT to the districts to conduct rapid risk assessments and report within the first 12 h of deployment followed by subsequent updates as the outbreak situation evolved. The NTF adapted and used the WHO EVD preparedness checklist to assess the districts’ level of preparedness. The checklist is composed of 11 key components and core capacities and tasks for both districts and health facilities that are required for a district to be operationally ready [10]. Through face-to-face interviews with key informants and discussions with the DRRT, the NRRT scored activities under each core capacity of preparedness. The assessment team verified responses by looking at documented evidence and implementation of the recommended core capacities at the district and health facility level. The NRRT held group discussions with health facility teams on epidemiological surveillance, case management, IPC, laboratory and contact tracing. Scores were assigned to each of the core capacity components (green for complete/in place and red for incomplete/not yet in place) and then percentages of the total tasks that were completed for each core capacity were computed for each district. Any district with a level of preparedness component at ≤50% was considered unprepared (red), between 50 and 75% was considered as fairly ready (yellow), and those above 75% were considered prepared for that component. The NRRT used the assessment results to identify capacity gaps for additional support to strengthen alert and response in the 30 high and moderate risk districts.

### Risk mapping

To establish DRC population movements and patterns across the DRC-Uganda border, the Population Connectivity across Borders (Pop-CAB) tool developed by US CDC was used to document, record, analyze, and map risks associated with population movements across borders. The NRRT held key informant interviews with the District Health Team (DHT), which included the District Health Officers, Surveillance Focal Persons, Health Inspectors and Health Educators, and local leaders at known border PoEs and along priority porous borders. Several official and non-official high-risk crossing points for population movement from DRC across border districts were identified. Trained facilitators also interviewed Township Chairpersons, Town Clerks, Sub-county Chiefs, Mayors, and transport operatives in the high-risk locations. In addition, Focus Group Discussions (FGDs) with village leaders, community health workers in the cited high-risk locations for DRC population were held. The FGDs were guided by an adapted PopCAB FGD guide to gather information about the type of people crossing the border, their points of origin, congregation points, reasons for entering Uganda, duration of their stay and destinations. GPS coordinates for each of the locations were recorded. We developed summaries from key informant interviews and focus group discussions aided with sketch maps to track cross-border movements and indicate key locations for the DRC population. The facilitators integrated participatory mapping with spatially-accurate, printed maps in the key informant interviews and focus group discussions to identify location information for priority points of interest and travel routes. Respondents described population movement patterns associated with refugees who leave the Uganda settlements to visit their relatives in DRC and return back to Uganda. In addition, they described the patterns of persons newly-arriving from the DRC to established refugee reception centers and resettlements areas in Uganda such as Kyangwali in Hoima District or Kyaka II in Kyegegwa District, as well as DRC traders traveling across Lakes Albert and George to major markets in towns in the Ugandan border and non-border districts for business, and others who fly directly to Entebbe International Airport from Goma, Eastern DRC. The DRRT used the results to identify several official and non-official high-risk border locations with population movement from DRC into border districts. These exercises revealed multiple DRC travelers’ destinations to towns as far as Uganda’s capital city of Kampala.

A mapping exercise was conducted by the International Organization for Migration (IOM) to establish the numbers, locations, and traveler flow of the numerous non-official border crossing points that exist between the DRC and Uganda through which people travel for social, business, and health reasons. This enabled selection of relevant PoEs for the establishment of screening, isolation and notification systems. Some of the busy border PoEs receive about 5000 people daily; however, the numbers increase tremendously and can reach over 20,000 people during market days, and this increases workload on screeners.

### The national EVD response plan and budget

With support from development partners and relevant stakeholders, the NTF developed the National EVD Contingency Plan to address the identified gaps in preparedness, and to support timely detection, response, and immediate containment of a potential EVD case in Uganda. This plan was developed in line with guidance provided in the International Health Regulations (IHR) 2005 for countries to develop core capacities to prevent, protect against, and rapidly respond to public health threats, including EVD. The plan was developed based on the rapid assessment preparedness reports, past experiences, and lessons learnt during the previous EVD outbreaks in Uganda [11]. The plan was also developed based on the One Health approach to Global Health Security at both national and subnational levels. This approach requires coordination across multiple sectors of government including human and animal health, agriculture, wildlife, water and environment, security, immigration and law enforcement. The NTF engaged several agencies including the Ministry of Health, Ministry of Agriculture Animal Industry and Fisheries (MAAIF), Ministry of Water and Environment (MWE), Uganda Wildlife Authority (UWA) under the Ministry of Tourism, Wildlife and Antiquities (MTWA), Ministry of Internal Affairs (MoIA), Ministry of Security (MoS), and the private sector among others. The EVD preparedness and response plan highlighted objectives, strategies, specific activities, and a performance and monitoring framework for each of the 11 components of preparedness and was costed accordingly. It highlighted a package of interventions for the districts in the 3 risk categories.

### Cross-border collaboration

After several cross-border contacts were made and points of entry coordination exchanges took place at local levels, the NTF and MoH engaged with the DRC Ministère de la Santé Publique (Ministry of Public Health) to convene a cross-border health meeting. Attendees included the East African countries at risk of introduction of EVD cases from the DRC in October 2018. Following this meeting, a bilateral ministerial meeting was held in Goma, DRC, in December 2018. The first meeting was designed to formalize cross-border collaboration and local-level exchange of information between affected countries, meanwhile the second meeting was meant to result in developing and signing of the Uganda-DRC Memorandum of Understanding (MoU) on cross-border collaboration for public health preparedness and response. The MoU enabled establishment of four surveillance zones between Uganda and the DRC.

### Surveillance

The NRRT trained DHTs, health and non-health frontline workers including members of security forces, Village Health Teams (VHTs) and political leaders on EVD case definitions and distributed copies of case investigation forms and contact tracing guidelines in assessed health facilities. Health facilities formed surveillance teams to heighten surveillance and active case search in order to detect any alert or suspect cases. Volunteers were trained on EVD screening at border PoEs and refugee reception centers with support from various partners. A PoE coordination committee developed a PoE toolkit which was approved by the NTF and adapted for use by MoH. The PoE teams screened everyone crossing into Uganda from DRC with infrared thermometers for body temperature, including all refugees at reception centers. Persons found with elevated body temperatures (> 38 degrees Celsius) were further screened for Ebola-like symptoms.

**Fig. 5:**
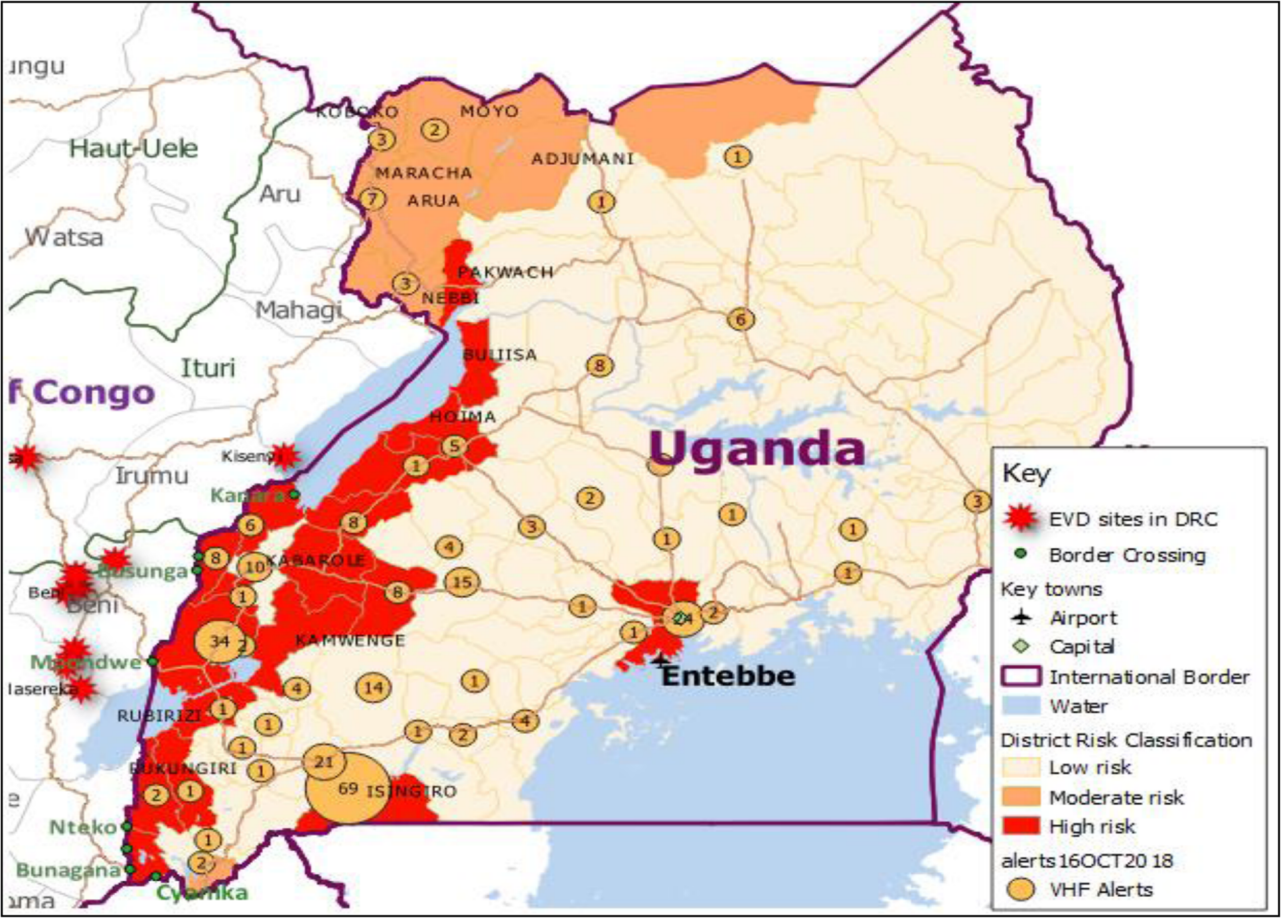
Map showing locations of verified EVD alerts in Uganda, March 2019

The NRRT trained laboratory health workers on specimen collection from suspected EVD cases and triple packaging for transportation to the Uganda Virus Research Institute (UVRI) for testing. With support from partners, ambulances were stationed in each of the 20 category 1 high-risk districts for rapid transportation of specimens to the UVRI Viral Haemorrhagic Fever (VHF) Program laboratory in Entebbe, ensuring delivery of samples within 24 h of collection. In addition, the MoH conducted a pilot of an electronic system to track collected samples to improve the monitoring of laboratory results turnaround time. Other actions undertaken in the preparedness period include modifications of the specimen collection, sample chains of custody and packing list forms. These modifications aimed to strengthen the existing biosecurity mechanisms of the National Laboratory Services. By end of May 2019, 641 Ebola alerts were verified countrywide (Fig. 5).

Although up through 9 June, no specimen collected from an alert tested Ebola positive, other VHF pathogens were detected in samples from 25 alert cases including Crimean Congo Haemorrhagic Fever [12] and Rift Valley Fever [13].

### Case management

The DTFs identified clinical staff including clinicians and community health workers whom the NRRT trained on EVD case definitions, transmission, clinical presentation, community surveillance, reporting, IPC, and case management including dignified safe burial. The trainings included simulation exercises such as donning and doffing of EVD PPE for case management and safe burial. During the trainings, the NRRT disseminated EVD guidelines and Standard Operating Procedures (SOPs) for patient management and, safe and dignified burial.

The NRRT also helped the DTFs to designate and set-up EVD isolation units at specified hospitals and health facilities ready to provide care to EVD patients. With support from partners, MoH constructed ten Ebola Treatment Units (ETUs) at designated hospitals in high-risk districts.

The NRRT conducted IPC assessments and instituted hand-washing and disinfection measures and positioned hand-washing stations at major health care facilities, official and high-risk unofficial PoEs, refugee transit and reception centers, and other relevant public places in the 30 high and moderate risk districts. With support from partners, MoH trained district-based IPC trainers who trained and mentored all relevant health facility workers on IPC practices in handling suspect, probable and confirmed EVD patients including safe waste management, and safe dignified burials.

### Logistics and supplies

The NTF Logistics Sub-committee conducted logistics assessments in at least five of the high-risk districts to determine a baseline, and then used the data for forecasting and quantification for medical, IPC, PPE, and laboratory needs for the districts. With support from the NRRT, the DTFs developed emergency contingency plans and budgets to facilitate EVD preparedness activities, which were reviewed and approved by the NTF. The MoH with support from partner agencies purchased and distributed PPE and other relevant supplies to all designated district hospitals and health facilities for case management. A number of development partners and international agencies supported the MoH financially to avail the preparedness logistics and supplies to the districts.

### Risk communication

The NTF generated a press statement which was read by the Minister of Health on 3rd August 2018 to inform the public about the EVD outbreak in DRC and increase alertness for surveillance in the 30 high- and moderate-risk districts and the rest of the country. The NTF Risk Communication Subcommittee developed subsequent press releases for the Minister of Health, The committee also developed the risk communication plan, radio spot messages, reviewed, printed and translated IEC materials which were translated in fifteen most spoken local languages in the 30 high-and moderate-risk districts across the western border of Uganda to ensure reach to the general public. Information specified signs and symptoms of EVD, the need to seek urgent medical care in case symptoms arise, and the importance of safe burial of the dead. EVD information was disseminated regularly through local newspapers, popular radio, and television stations. In addition, the DTFs disseminated IEC on EVD through regular radio talk shows, spot messages, and posters in high-risk communities. They also supported trained community volunteers to carry out communal and door-to-door EVD health education.

### Vaccination

**Fig. 6:**
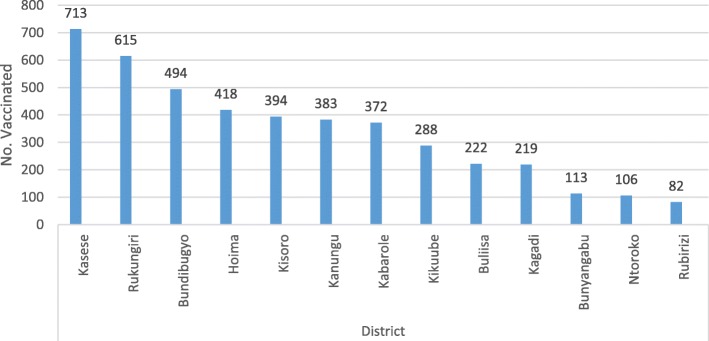
Number of health workers vaccinated in 13 high risk districts of Uganda, April 2019

The NTF sought ethical and institutional approval for prophylactic use of the rVSV-ZEBOV vaccine currently used in the DRC based on its demonstrated protective efficacy against the Ebola virus-Zaire type. This particular vaccine was being administered in DRC and had demonstrated positive protective results and potency against the Ebola virus-Zaire type [12]. The aim of the initial Ebola vaccination campaign was to protect frontline health and non-health workers in Uganda under a compassionate-use strategy to protect persons at potential risk for EVD in advance of an outbreak. Once approval was granted, the MoH, with support from the WHO, secured 3000 vaccine doses, established and trained national vaccination teams, organized cold chain logistics, selected districts and most-at-risk workers in health care facilities, PoEs and other locations (laboratories, airport, etc.), and commenced voluntary vaccination. Vaccination proceeded according to a site-by-site plan ranking highest- to lowest-risk sites for EVD case introduction from the DRC. The vaccination campaign planning committee used PopCAB results from identified districts to inform their decisions about which heath care facilities and Poe to target during the initial vaccination campaign. An additional 3000 doses of vaccines were later secured to scale up the vaccination exercise. By the end of April 2019, Uganda had vaccinated 4419 health workers in 13 high-risk districts (Fig. 6.

The vaccination team in their wisdom selected health workers they considered more at risk compared to others and targeted those for vaccination rather than vaccinate all health workers. This was the first time Ebola vaccination was conducted as part of a country’s preparedness response.

### Full-scale simulation exercise

A WHO Joint Monitoring and Assessment of EVD preparedness in Uganda conducted in December 2018 showed significant progress, with up to 92% readiness nationally. The MoH therefore recommended a full-scale simulation exercise to test the operational capabilities of all components of the preparedness and response plan.

A full-scale simulation exercise (FSX) was conducted in April 2019 to test the preparedness and response systems at three levels of operation: community, district, and national. The exercise was conducted in a highly stressful environment, simulating actual response conditions. The exercise simulated three suspected cases of EVD. The first case tested the response systems at a designated land PoE in Kasese District (Mpondwe Border Point), and referral to an Ebola Treatment Unit (ETU), while the second came through Entebbe International Airport. The third case tested community surveillance and reception of an EVD suspected case in a non-ETU facility in Kasese District, and subsequently safe and dignified burial when the patient died. The coordination structures at national and district levels were also tested.

In all scenarios, detection of a suspected EVD case was timely, though reporting from the community to the national level was delayed. The simulation exercise identified strengths and gaps in the development and implementation of preparedness and response measures. The exercise also identified areas for improvement in coordination of the response at district level, safe referral of EVD suspected cases, infection prevention and control, and management of severely ill patients. It was recommended that preparedness efforts prioritize skilling health care providers through intensified supervision, mentorships and drills.

### Key challenges

The conflation of political instability and rebel activities coupled with community mistrust in the outbreak response in Eastern DRC have made it very difficult for the DRC and its partners to control what has now become the second-largest and -longest EVD outbreak recorded in history [14]. As a result, the outbreak continues to rage on and on many months after it was declared, and the number of confirmed EVD cases in the DRC have been increasing, with new cases being declared closer to the Ugandan border [15]. At the same time, Uganda continues to receive a large influx of people from DRC through official and unofficial PoEs, posing a huge strain on the border screening efforts put in place. Moreover, EVD preparedness efforts are showing multiple signs of fatigue from all sides and is thus becoming increasingly hard to sustain, with funding and resources still inadequate to cover all high-risk districts, and yet the need to sustain the preparedness momentum at both national and district levels is greater than ever.

## Discussion

A day after the official declaration of the 10th EVD outbreak in the DRC, Uganda’s MoH initiated a quick EVD preparedness response by activating the NTF and PHEOC to a response level and mandating its members and partners to plan, mobilize resources and coordinate implementation of the full range of EVD preparedness pillar activities. With support from several partner organizations, Uganda was able to activate the NTF and create an active IMS and a partner coordination matrix, mobilize resources and carry out preparedness activities in 30 designated high- and moderate-risk districts on its western border with DRC as well as on adjacent borders with Rwanda and South Sudan and the central urban hubs of Kampala and Entebbe, linked to population movements from and to the DRC’s outbreak hotspots. In addition, Uganda has established multi-sectoral and multidisciplinary national and district task forces, and technical subcommittees for each preparedness pillar that advise the NTF and act as liaisons between the NTF and the high- and moderate-risk districts. These quick actions were most influenced by Uganda’s experiences with four previous EVD outbreaks [16]. With this experience and support from partners, activation was quick and was established with relative ease. The lasting impact of these efforts is a high likelihood that it could generate momentum towards national and partners’ commitments to support long-term emergency preparedness for future outbreaks.

The WHO recommends EVD preparedness activities in countries with close proximity to EVD epicenters to enable them respond should there be any importation of EVD cases. Uganda is among nine priority countries neighboring DRC since the present 10th EVD outbreak started [17]. After the 2014 EVD outbreak in West Africa, the WHO proposed an agenda for changing its strategy in the face of epidemic and pandemic threats, which has been largely accepted by WHO Member States, including Uganda. Through this agenda, WHO was able to support Uganda with a quick, proactive, result-driven, resourced and well-equipped team. Indeed, Uganda, supported by its internationally recognized National VHF Program at UVRI, was the first neighboring country in the WHO-AFRO region to initiate and engage in a fully-fledged emergency preparedness and response program, which also included prophylactic use of Ebola vaccination.

The preparedness assessment found that all 30 high- and moderate-risk Ugandan districts had less than a 50% score for EVD readiness at baseline. It also found that the average score for EVD preparedness was highest in laboratory and lowest in budgets, safe burials, and contact tracing. These findings were similar to WHO’s initial assessments done across EVD African countries neighboring DRC during the 2018 EVD outbreak as well as what was found by WHO in the 2015 EVD preparedness assessment for west African countries (23). The UNHCR, IOM and Medical Teams International (MTI) that provide active health, border, and mobility care for refugees in the settlements had already put in place measures to strengthen EVD preparedness such as EVD trainings, EVD screening, IPC measures, and dissemination of EVD IEC materials.

Following the IDSR guidelines, the national surveillance system was strengthened with training of all national and district surveillance focal persons and facilitation for timely detection, reporting, and investigation of EVD alerts with focus on cross-border surveillance and early patient triage at high-risk referral health care facilities. The EOC continues to provide timely information about disease epidemics while the NTF closely continues to guide, monitor, and supervise the preparedness activities. The country has built capacity and established a good laboratory network within districts, regional referral hospitals, and at the national level to collect, package, store and transport samples to the National VHF Program laboratory at UVRI. The VHF Program laboratory at UVRI is a regional diagnostic referral laboratory for VHF and routinely screens all suspect specimens for Ebola (Bundibugyo, Sudan), Marburg, CCHF and RVF. There is also well-developed local capacity for social mobilisation during epidemics with engagement of political, local and religious leaders. Many health workers have been trained in EVD case management and provided with updated guidelines and SOPs with support from several partner agencies.

Nine months after the declaration of the 10th EVD outbreak in the DRC, Uganda continues to conduct preparedness activities to prevent introduction and spread of EVD into the country. While WHO continues to support this effort, the duration and extent of this outbreak has come as a surprise to many. As a result, governments, donors, and partners have all been stretched to a point where the preparedness strategy needs to be revisited. Specifically, it is important to redefine strategies in order to both maintain the readiness momentum at a time where the risk of outbreak spillover has increased, as well as to plan for the human, technical, and financial resources needed to balance this protracted effort with the rest of the national health security agenda. This agenda requires at least equal time, efforts, and resources as EVD preparedness, yet the capacity of an at-risk country like Uganda is limited and cannot address both needs fully. A WHO meeting on Strengthening Partnership for Improving Ebola Preparedness and Readiness took place at the end of April 2019 and this issue was addressed.

### Next steps

As the EVD outbreak continues in DRC, the risk of cross-border transmission remains imminent. Subsequently, the country has plans to expand and intensify preparedness activities to additional districts, with a special focus on the use of universal precautions and ways to strengthen and sustain IPC practices at all health facilities in high-risk districts. The broad EVD preparedness workgroup continues to strengthen cross-border collaboration and to engage the community in door-to-door risk communication and surveillance in all high-risk districts. There is also need to accelerate vaccination of at-risk frontline health workers in the other high-risk districts. The MoH, with support from partner agencies, has started to conduct drills and simulation exercises in the most at-risk districts bordering the DRC to evaluate preparedness and response capacity. The MoH also plans to improve sample transportation and re-establish a national border health unit that has been inactive for many years.

## Conclusions

Within the first month of declaration of the 10th EVD outbreak in DRC, Uganda’s MoH had established a strong coordination and surveillance system to effectively alert and respond to any EVD suspect case and to mitigate risk of importation of EVD into Uganda. With continued technical and financial support from many partners, the country has strengthened EVD preparedness and timely detection, investigation and response to EVD alerts. Sustained efforts are required to support refreshing of health workers, provision of infection control supplies, maintenance of infrastructure, provision of equipment for case referral and isolation, regular drills and simulation exercises in key technical areas, continued risk communication, community engagement, resource mapping as well as countrywide coordination.

### Addendum

On June 10, Uganda detected its first cases of EVD as a result of a spillover event from the DRC. While the details of the cases will be presented in another paper, cases were quickly recognized, specimens safely collected and efficiently transported for diagnostic testing and confirmation at UVRI. Case patients were rapidly relocated to an ETU for isolation and care. As of 22 June 2019, no secondary transmission from these three cases have occurred in Uganda, a clear demonstration of the effectiveness of the Uganda preparedness program.

## Data Availability

The data sets and the reports that support this write up belong to the MoH Uganda. Due to confidentiality reasons, the data sets and reports are not publically available. However, the data sets and the reports could be availed upon reasonable request and with permission from the MoH Uganda.

## Abbreviations

AFENET: African Field Epidemiology Network
Africa CDC: Africa Centres for Disease Control and Prevention
China CDC: Chinese Centre for Disease Control and Prevention
DFID: Department of International Development
DHO: District Health Officer
DHT: District Health Team
DRC: Democratic Republic of Congo
DRRT: District Rapid Response Team
DTF: District Task Force
ETU: Ebola Treatment Unit
EVD: Ebola Virus Disease
FAO: Food and Agriculture Organisation of the United Nations
FGD: Focus Group Discussion
GHSA: Global Health Security Agenda
IDI: Infectious Diseases Institute, Uganda
IHR: International Health Regulations
IMC: Incident Management Commander
IMS: Incident Management System
IOM: International Organisation for Migration
JICA: Japan International Corporation Agency
JMEDICC: Joint Mobile Emerging Disease Intervention Clinical Capability
KOICA: Korea International Corporation Agency
MakSPH: Makerere University School of Public Health
MoH: Ministry of Health
MSF: Medecins Sans Frontieres (Doctors Without Borders)
MTI: Medical Teams International
MUWRP: Makerere University Walter Reed Project
NRRT: National Rapid Response Team
NTF: National Task Force
PHEOC: Public Health Emergency Operations Centre
PHFP: Uganda Public Health Fellowship Program
PoE: Point of Entry
UNDP: United Nations Development Program
UNHCR: United Nations High Commissioner for Refugees
UNICEF: United Nations Children’s Fund
URCS: Uganda Red Cross Society
USAID: United States Agency for International Development
US-CDC: United States Centers for Disease Control and Prevention
UVRI: Uganda Virus Research Institute
VHF: Viral Haemorrhagic Fever
VHT: Village Health Team
WFP: World Food Program
WHO: World Health Organisation
